# An Antioxidant Screen Identifies Candidates for Protection of Cochlear Hair Cells from Gentamicin Toxicity

**DOI:** 10.3389/fncel.2017.00242

**Published:** 2017-08-18

**Authors:** Volker Noack, Kwang Pak, Rahul Jalota, Arwa Kurabi, Allen F. Ryan

**Affiliations:** ^1^Department of Surgery and Otolaryngology, School of Medicine, University of California, San Diego, La Jolla CA, United States; ^2^VA San Diego Healthcare System, San Diego CA, United States

**Keywords:** inner ear, sensory cell, redox, ototoxicity, damage prevention, hair cell, screen

## Abstract

Reactive oxygen species are important elements in ototoxic damage to hair cells (HCs), appearing early in the damage process. Higher levels of natural antioxidants are positively correlated with resistance to ototoxins and many studies have shown that exogenous antioxidants can protect HCs from damage. While a very wide variety of antioxidants with different characteristics and intracellular targets exist, most ototoxicity studies have focused upon one or a few well-characterized compounds. Relatively little research has attempted to determine the comparative efficacy of large variety of different antioxidants. This has been in part due to the lack of translation between cell culture and *in vivo* measures of efficacy. To circumvent this limitation, we used an *in vitro* assay based on micro-explants from the basal and middle turns of the neonatal mouse organ of Corti to screen a commercial redox library of diverse antioxidant compounds for their ability to protect mammalian HCs from a high dose of the ototoxic antibiotic gentamicin. The library included several antioxidants that have previously been studied as potential treatments for HC damage, as well as many antioxidants that have never been applied to ototoxicity. The micro-explants were treated with 200 μM gentamicin alone, gentamicin plus one of three dosages of a redox compound, the highest dosage of compound alone, or were untreated. HC counts were determined before the gentamicin insult and at 1, 2, and 3 days afterward to evaluate the HC survival. From a total of 81 antioxidant compounds, 13 exhibited significant protection of HCs. These included members of a variety of antioxidant classes with several novel antioxidants, not previously tested on HCs, appearing to alleviate the damaging gentamicin effect. Some compounds previously shown to be protective of HCs were correspondingly protective in this *in vitro* screen, while others were not. Finally, one of the three pro-oxidant compounds included in the library as well as six antioxidants exhibited evidence of toxicity in the absence of gentamicin. The results demonstrate the wide variability in the ability of antioxidants to protect HCs from high-dose gentamicin damage, and identify promising candidate leads for further study as potential drug targets.

**Highlights:**

• A medium-throughput assay based on micro-explants of the organ of Corti was developed to screen mammalian cochlear hair cells for protection from damage by ototoxins.
• Eighty one antioxidants and 3 pro-oxidants were evaluated for hair cell protection from high-dose gentamicin.
• Thirteen antioxidants were significantly protective, while 6 proved to be damaging.
• The use of a common assay permitted an evaluation of the relative capacity of different antioxidants for the protection of hair cells.

## Introduction

Ototoxicity, hearing loss and vestibular disorders are significant side effects of a number of valuable medications. This includes important categories of drugs used to treat life-threatening illnesses, such as aminoglycoside antibiotics and platinum-based anti-neoplastic agents. Hearing loss due to aminoglycosides is estimated to occur in almost 50% of patients (Fausti et al., 1992), while the incidence of hearing loss following cisplatin or carboplatin treatment can be as high as 75–100% (McKeage, 1995; Rybak et al., 2009). The potential for ototoxicity can limit the use of these drugs. If their use is unavoidable, it can result in hearing loss up to and including complete deafness. Vestibular disorders are also a common side effect (Schacht et al., 2012).

The most vulnerable elements of the inner ear to ototoxic drugs are the sensory HCs (Wong and Ryan, 2015). The cellular mechanisms that underlie HC damage are incompletely understood. However, there is extensive evidence that ototoxins induce the formation of ROS as an early step in the damage course. ROS formation precedes visible damage to the cell by up to 24 h (h) (Choung et al., 2009), consistent with an early role. The antioxidant glutathione increases in HCs from the base to the apex of the cochlea (Sha et al., 2001a), while sensitivity to ototoxins decreases from base to apex (e.g., Ryan and Dallos, 1975). In animals, treatment with antioxidants or the upregulation of antioxidant genes has been convincingly shown to delay or prevent ototoxin-induced HC loss (e.g., Garetz et al., 1994a,b; Rybak et al., 1999; Sha et al., 2001b).

This success in animal experiments has led to a limited number of clinical trials that have evaluated the effects of antioxidant treatment on ototoxic or noise-induced HC and hearing loss. Kocyigit et al. (2015) reported that NAC provided a degree of protection against amikacin ototoxicity at the highest frequencies tested. A trial of NAC in military trainees undergoing firearms training also showed some degree of protection (Lindblad et al., 2011). In contrast, a recent trial of NAC versus placebo treatment prior to stapedectomy, where drilling noise and surgical trauma can produce sensorineural hearing loss, showed an equivalent level of hearing loss (∼10 dB) in both groups, and thus was unable to demonstrate a protective effect (Bagger-Sjöbäck et al., 2015). Kramer et al. (2006) found no effect of NAC on temporary threshold shift induced by loud music.

The reasons for the variability in clinical trial results are unclear. However, the degree of experimental control in clinical trials is much less than that in animal studies. Another possibility is that the antioxidant dose actually reaching the cochlea after systemic administration may be non-optimal. Moreover, different antioxidants can exert their effects via several distinct mechanisms and targets. This includes scavenging the radical species that initiate peroxidation, quenching singlet oxygen, chelating metals, breaking free radical chain reactions, reducing the concentration of O_2_, preventing oxidation of proteins or DNA, and/or stimulating endogenous antioxidant enzymes (Lü et al., 2010). Because antioxidants may employ one or more of several mechanisms, differences in their effectiveness may vary with differences in the cellular processes involved.

It should also be noted that while many antioxidants have been tested for their ability to protect HCs, there are many other compounds with antioxidant properties. Moreover, few studies have compared HC protection by antioxidants in a standardized model, so that relative effectiveness can be estimated. For the reasons noted above, differences in their potential for HC protection seem likely. Hence, a comparative screen of a large number of antioxidants using a standardized model of HC damage could identify novel antioxidants with protective properties and might also provide evidence regarding differences in protective efficacy.

To address these issues, we screened a commercial library of antioxidants in a single model of aminoglycoside-induced HC damage. Micro-explants from the neonatal murine oC were exposed to a high dose of the ototoxic aminoglycoside gentamicin to elicit oxidative stress. We used a transgenic mouse line in which HCs express GFP under the control of a HC-specific promoter (Masuda et al., 2011). This allowed for visualization of HC loss over the course of gentamicin treatment in culture. HC loss was compared between micro-explants without any treatment, with gentamicin treatment alone, or with gentamicin and antioxidant co-treatment. This allowed us to determine the relative efficacy of the 81 different antioxidants and 3 pro-oxidants to influence HC viability.

## Materials and Methods

### Animals

Experiments were performed on transgenic animals in which eGFP was selectively expressed in HCs under the control of a *pou4f3* promoter construct (Masuda et al., 2011). All experiments were performed to National Institutes of Health guidelines and approved by the Institutional Animal Care and Use Committee of the VA San Diego Medical Center.

### Micro-Explant Preparation

The oC was dissected from the cochleas of postnatal day 3–5 *pou4f3*/eGFP mouse pups. The apical region of each epithelium, which is relatively insensitive to aminoglycoside toxicity, was discarded. The basal and middle regions of the epithelium were divided using a diamond scalpel into micro-explants consisting of approximately 20 inner HCs and the 60 associated outer HCs. Micro-explants were individually plated in each well of a flat-bottom 96 well plates in media consisting of DMEM F-12 (Gibco) plus 30 U/ml Penicillin and 5% FBS, maintained in a humidified tissue culture incubator (37°C, 5% CO_2_).

### Redox Library Screening

Screening was performed using the Screen-Well Redox Library (BML-2835, Enzo Life Sciences, Farmingdale, NY, United States). The library consists of 81 antioxidant and 3 pro-oxidant compounds. The library represents a variety of classes of compounds, including some that have been shown previously to protect HCs from damage. This includes glutathione (Garetz et al., 1994a), α-lipoic acid (Rybak et al., 1999), α-tocopherol (Fetoni et al., 2003), D-methionine and ebselen (Kopke et al., 1997). Many pharmacological compounds that had not been previously applied to HC protection were also present, providing the opportunity to identify new protective compounds discovery and repurposing. The compounds present in the library are listed in Supplementary Table 1. Test compounds were initially dissolved in DMSO and diluted in culture media with the total amount of DMSO adjusted to a final concentration of 0.1%.

Each experimental oC micro-explant was pretreated for 24 h with one of the library compounds at concentration of 10, 100, or 1000 μM, performed in triplicate wells. The next day, the media were withdrawn, fresh media containing 200 μM gentamicin plus the pharmacological compound with the appropriate concentration was added, and the micro-explants were cultured for 72 h. Untreated (negative) controls were maintained in media alone and positive controls were treated with 200 μM gentamicin alone. Media for both control groups contained 0.1% DMSO, to match the experimental groups. Compounds were screened in duplicated 96-well plates with seven compounds per plate, plus controls. The size of the testing plates and performance of experiments on different days and with different media or gentamicin batches required replication of both control conditions on each plate, for comparison with the results from the compounds evaluated on that plate.

Green florescent protein-positive HCs were imaged by fluorescence microcopy on each day of treatment, and survival curves were generated for each compound and condition. HC counts, including both inner and outer HCs, were evaluated in ImageJ, and normalized as percentages to the number of HCs present on D1, prior to the start of gentamicin treatment. Any micro-explants that did not attach and flatten in the well by D1 were excluded (usually less than 3% per plate), because HC counts could not be accurately quantified at that time. There were sufficient wells on each plate that three micro-explants per condition could almost always be accommodated even with some unattached samples.

### Statistical Analysis

Statistical analysis was performed using GraphPad Prism 6, StatView 5, using the Kruskal–Wallis non-parametric ANOVA to detect treatment effects. Individual condition comparisons were performed using the Mann–Whitney *U* test, with correction for multiple comparisons. For purposes of the figures, standard deviations were calculated from the non-normalized HC counts.

### Validation

Redox compound “hits” were identified in the initial round of screening as deviating significantly from the controls. Following this initial identification, repeat plates were prepared in an identical manner for all hits, for a total N of 6 micro-explants. Statistical analysis was then repeated. Hits that demonstrated a repeatable effect in the following round were considered to be confirmed.

## Results

### Control Micro-Explants and Gentamicin Cytotoxicity

**FIGURE 1:**
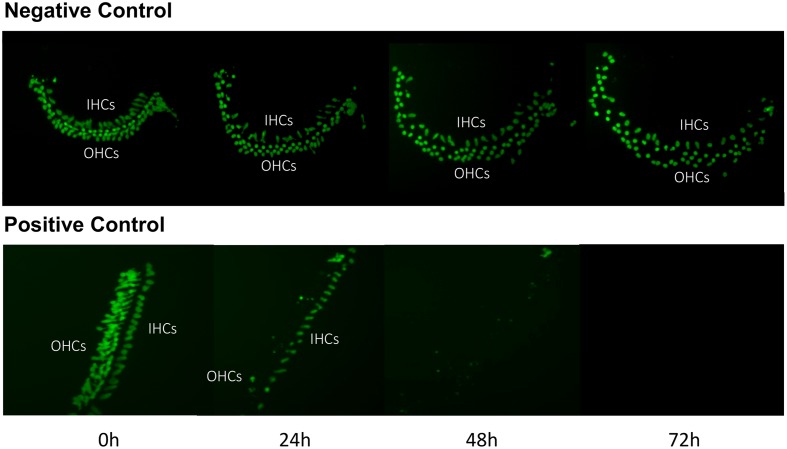
Control cultures. Montage of a typical negative control oC micro-explant **(top)** and a 200 μM gentamicin-treated positive-control micro-explant **(bottom)**, illustrating GFP-positive HCs from D1 (just prior to addition of gentamicin to the positive controls) to D4. In the negative control, there is modest loss of HCs, primarily on D3 and D4. In the positive control, HC loss is significant by D2 and near-total on D3 and D4.

**FIGURE 2:**
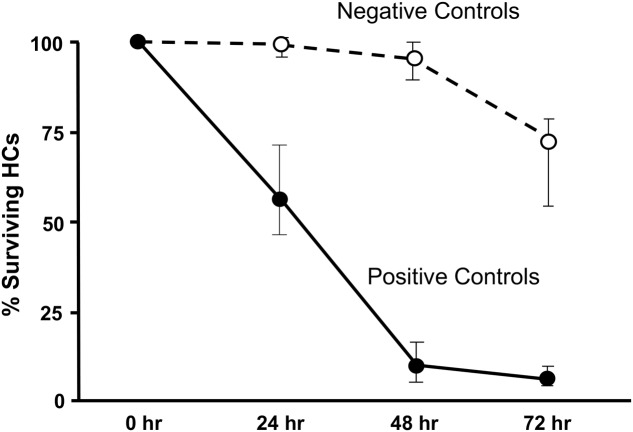
Control culture cell counts. All of the positive and negative control oC micro-explants from all plates were counted and the results pooled to illustrate the control survival curves between D1 and D4. Data points represent medians, and error bars present the interquartile range.

Imaging of GFP-positive HCs in control wells typically showed HC survival similar to that illustrated in **Figure 1**. Untreated (negative control) micro-explants maintained only in culture media showed near-complete HC survival from D1-D3, with some HC loss on D4. In contrast, HCs treated with 200 μM gentamicin (positive control) showed significant losses by D1, and severe losses by D2 and D3. HC counts from negative and positive control micro-explants in each plate were generated, converted to percent survival relative to Day 1 (D1, just prior to gentamicin exposure), and averaged across all plates. The results are illustrated in **Figure 2**. Negative controls showed high levels of HC survival on both D2 (96%) and D3 (93%). By D4, negative control micro-explants showed somewhat reduced HC survival (71%). Positive control micro-explants showed significantly reduced survival on D2, after 24 h exposure to gentamicin (53%), and significantly lower survival on D3 after 48 h gentamicin exposure (14%). D4 gentamicin showed continued loss of GFP-positive cells (8%). As expected, Kruskal–Wallis non-parametric ANOVA and Mann–Whitney *post hoc* tests showed a highly significant difference between negative and positive controls from D2–D4 (*p* < 0.0001). It should be noted that variation in controls was noted, as can been seen in later figures. In particular, some negative controls showed HC loss on D3 greater than the average range, while some positive controls showed HC loss on D1 that was less than the average range. This is reflected in the greater variation shown in **Figure 2** for those time points. However, data were always compared statistically to positive controls from the same plate, which were generated with the same batches of media and gentamicin.

### Library Results

**FIGURE 3:**
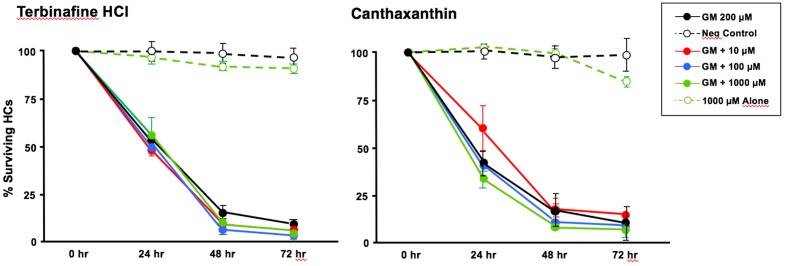
Normalized D1-4 HC survival curves for two antioxidants (canthatraxin and terbinafine HCl) that had no significant effect on normal or gentamicin-treated micro-explants. HC survival with 1000 μM of the compound alone (open green circles) was not significantly different from an untreated, negative control micro-explant (open black circles). Treatment with the compound at 10 μM (solid red circles), 100 μM (solid blue) or 1000 μM (solid green) plus 200 μM gentamicin was not significantly different from 200 μM gentamicin alone (solid black). *N* = 6 micro-explants per data point.

**FIGURE 4:**
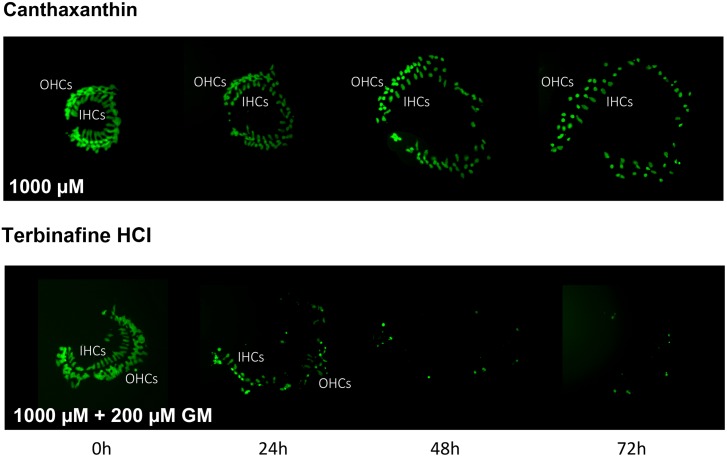
Montages illustrating representative oC micro-explants treated with 1000 μM canthatraxin alone, or 100 μM terbinafine HCl plus 200 μM gentamicin, for 72 h.

Of the 81 antioxidants and 3 pro-oxidants in the redox library, 68 antioxidants and 2 pro-oxidants had no effect on either untreated or gentamicin-treated micro-explants. Two examples of antioxidants with no effect are illustrated in **Figures 3, 4**. Micro-explants treated with the highest concentration of each antioxidant (1000 μM) exhibited HC counts very similar to that seen for negative control micro-explants cultured in media alone. Micro-explants treated with three concentrations of each antioxidant plus 200 μM gentamicin showed HC counts that were similar to that observed with gentamicin alone.

**FIGURE 5:**
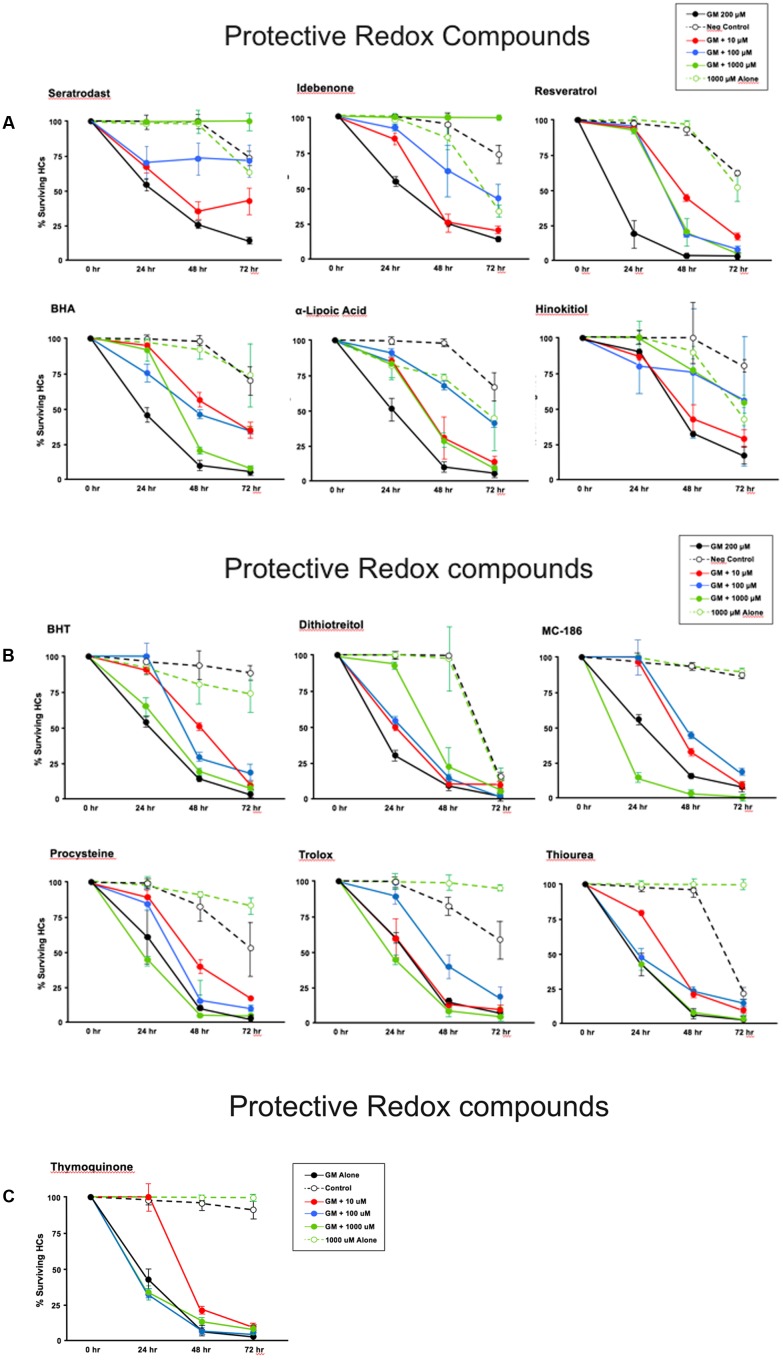
**(A–C)** Normalized D1–4 HC survival curves for 13 compounds that showed significant protection from gentamicin. The data also illustrate the variability seen in positive in negative controls. This variability necessitated the use of separate controls for each plate in our assay. *N* = 6 micro-explants/data point.

**FIGURE 6:**
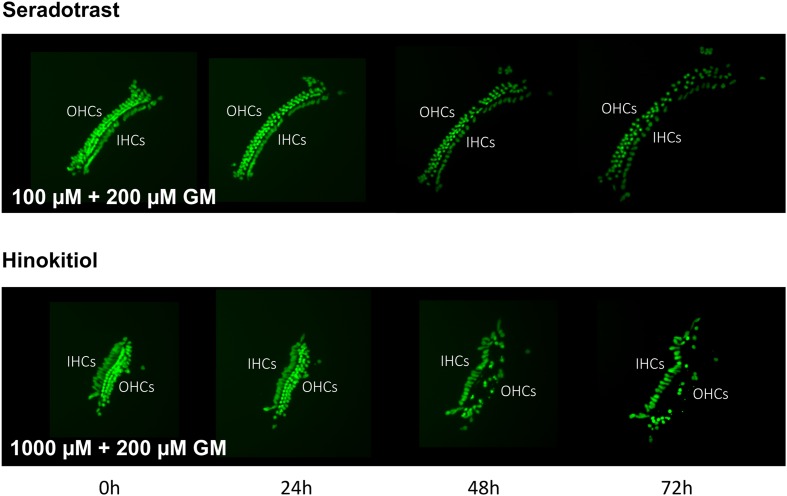
Montages illustrating representative micro-explants treated with 100 μM seratrodast plus 200 μM gentamicin or 1000 μM hinokitiol plus 200 μM gentamicin, for 72 h.

Thirteen antioxidants exhibited statistically significant protection of HCs in micro-explants treated with both the compound and gentamicin. The results for these compounds are illustrated in **Figure 5**, arranged in order of degree of HC protection. Seratrodast and idebenone exhibited the strongest protection from gentamicin toxicity, which was complete at 1000 μM, the highest dosage tested, and, also, significant at 100 μM. However, idebenone alone appeared to reduce HC survival on D4. Resveretrol, BHA, α-lipoic acid, hinokitiol, BHT, dithiotreitol and MC-186 were strongly protective at some gentamicin exposure times and antioxidant dose concentrations. However, at the highest concentration tested, MC-186 significantly enhanced early HC loss due to gentamicin. Procysteine and trolox were more modestly protective at some times and concentrations, while thiourea and thymoquinone were protective only at 24 h, and only for the lowest antioxidant concentrations. Examples of two micro-explants that were protected by antioxidants are presented in **Figure 6**.

**FIGURE 7:**
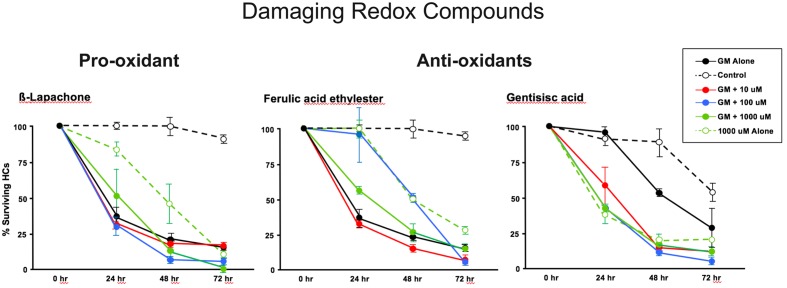
Normalized D1–4 HC survival curves for 1 pro-oxidant (β lapachone) and 2 representative antioxidants (ferulic acid ethylester and gentisisc acid). When each of these compounds was applied to micro-explants in the absence of concurrent gentamicin, far more HC damage was observed than in untreated (negative controls) micro-explants. *N* = 6 micro-explants/data point.

The pro-oxidant β-lapachone and the antioxidants disuliram, ferulic acid ethylester, gossypol, gentisisc acid and caffeic acid were significantly toxic to HCs in the absence of gentamicin, although none significantly worsened gentamicin-induced HC damage. HC survival curves for the pro-oxidant and two damaging antioxidants are presented in **Figures 7**. Image montages demonstrating the HC toxicity after treatment with lapachone alone, or ferulic acid ethylester alone, for 72 h, is seen in **8**.

**FIGURE 8:**
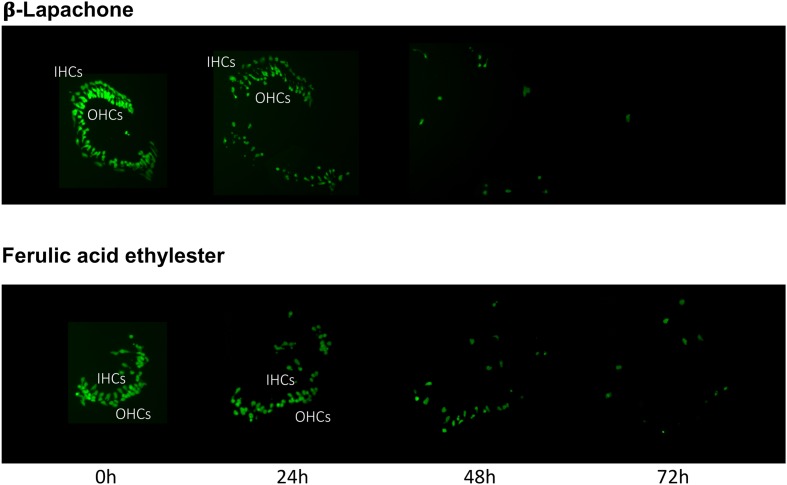
Montages illustrating representative oC micro-explants treated with 1000 μM β-lapachone alone, or 1000 μM ferulic acid ethylester alone, for 72 h, illustrating HC toxicity without gentamicin.

## Discussion

### Summary

We have developed an assay based on micro-explants of the neonatal mouse oC to screen a variety of antioxidants for their ability to alter aminoglycoside damage to mammalian cochlear HCs *in vitro*. All significantly positive “hits” were confirmed by re-screening. This resulted in the identification of 13 out of 81 antioxidants that offered significant protection against gentamicin-induced HC damage. In addition, we identified several antioxidants that showed evidence of toxicity in our assay, even in the absence of gentamicin. Some of the protective antioxidants identified in the screen have been studied previously and a protective role identified *in vitro* or *in vivo*. The HC protective role of others is identified here for the first time. The screen also provides comparative information on the protective capacity of antioxidants, under the conditions tested, with some antioxidants offering significant superior protection.

### Mammalian Organ of Corti Micro-Explant Assay

Many assays for the evaluation of compounds on HCs have been developed. The assay presented here, like any preclinical assay, has both advantages and disadvantages. A key advantage of the micro-explant screening assay is that it employs mammalian HCs rather than HCs from different animal classes or mammalian cell lines. This is important since mammalian HCs, and especially cochlear HCs, are quite different from the HCs of different animal classes such as birds or fish. The outer HC, the most vulnerable element in the mammalian cochlea, is not present in other classes of animals. HCs are of course quite different from mammalian cell lines. Thus, it might be argued that an assay based on the mammalian oC gives results more applicable to humans.

Another advantage of the model is the ability to evaluate the effects of a much larger number of compounds than can be achieved with an *in vivo* mammalian model. This is because several explants can be generated from each murine oC. While the assay is by no means high-throughput, it would allow the screening of a few hundred compounds. Because the assay is uniform, it allows not only hit identification, but also information on relative effectiveness. Thus, we found that seratrodast and idebenone were the most effective HC protectants under the conditions of this assay. Since seratrodast has not been studied as a HC protectant in the past, this compound, plus other novel antioxidants identified, deserve more extensive study.

Of course, there are also disadvantages to this assay system. Since adult HCs do not survive in culture, the assay is based on neonatal HCs that are not yet functionally mature. They may respond differently to gentamicin or to antioxidants than adult HCs. In addition, the number of compounds that can be tested is limited. Thus, screening very large compound libraries is beyond the capacity of our method. Similarly, including a very large number of conditions, such as a large range of gentamicin dosages, would be difficult when also varying compound concentrations. Finally, this is a screening assay, which as with all screens does not provide definitive data, but rather identifies candidates that warrant further study. These limitations must be considered when interpreting the results of the assay.

### HC Protective Antioxidants

A relatively small number of antioxidants among those tested proved to be protective to HCs. The range of protection varied from nearly complete to modest. There was no single category of antioxidant that proved to be superior to others. Several different categories of antioxidants were represented in the protective compounds. Moreover, often some antioxidants of the same class were found to have no effect on HC survival, or even in a few cases to be harmful. Hence, the mechanism of protection is believed to be associated with their antioxidant capacity but is not fully understood.

#### Seratrodast

Seratrodast is a quinone antioxidant, and was one of the most protective compounds identified in the screen. It has not previously been studied as a protective agent for HCs. Seratrodast acts as a free radical scavenger. It is not only an antioxidant, but also a blocker of the thromboxane A2 receptor and is used in the treatment of asthma. While thromboxane A2 has been implicated in vascular disorders of the inner ear (e.g., Umemura et al., 1993), it has never been associated with ototoxicity. It therefore seems likely that the protective effects of seratrodast are related to its antioxidant properties.

#### Idebenone

Idebenone, is a quinone antioxidant and a synthetic analog of co-enzyme Q. It is a free radical scavenger that was also highly effective in protecting against high-dose gentamicin-induced HC damage in the assay. Sergi et al. (2006) found that systemically administered idebenone provided protection against noise-induced hearing loss in guinea pigs. Since this protection was not additive with that of α-tocopherol (vitamin E), it was assumed that their mechanisms of action were overlapping. There has been no previous study of idebenone as a HC protectant against ototoxins.

#### Resveratrol

Resveratrol is a naturally occurring stilbene polyphenolic antioxidant that acts as a free radical scavenger. It was very effective in protecting HCs from gentamicin toxicity. A number of studies have shown resveratrol to be protective against various forms of HC damage including cisplatin (Yumusakhuylu et al., 2012) and noise (Hanci et al., 2016) *in vivo*, as well as gentamicin *in vitro* treatment (Bonabi et al., 2008). In addition to its antioxidant properties, resveratrol has been shown to activate sirtuin-1 and to de-acetylate NFκB, and these properties have been implicated in the protection of HCs from anoxic damage (Wang et al., 2013).

### Butylated Hydroxyanisole

Butylated hydroxyanisole is a phenolic antioxidant and potent free radical scavenger that is often used as a food additive to prevent oxidative damage, especially to lipids. It was a very effective HC protectant against high-dose gentamicin. BHA has not previously been studied as a HC protectant. Reports that BHA is a carcinogen at high levels have since been repudiated, but they led to limits on permissible BHA levels and a decline in BHA use in some foods from the 1990s (Hui, 2006).

### DL-α-Lipoic Acid

DL-α-lipoic acid is a sulfur-containing antioxidant. It is a free radical scavenger and metal chelator that also enhances intracellular levels of glutathione (Shay et al., 2009). It was very protective of HCs against high-dose gentamicin. There have been numbers of studies of α-lipoic acid as a protective agent against cisplatin ototoxicity (see Rybak et al., 2009 for a review). Other animal studies have evaluated this compound as a protectant against age-related hearing loss (Ahn et al., 2008) and cochlear implant trauma (Chang et al., 2017).

#### Hinokitiol

Hinokitiol (β-thujaplicin) is a naturally occurring antioxidant, found in the heartwood of certain plants. It acts as a metal chelator and, also, enhances the activity of superoxide dismutase (Huang et al., 2015). It was found to be very protective in the assay. It has not been previously studied as a HC protectant. In addition to its antioxidant properties, hinokitiol has been shown to reduce inflammation via suppression of NFκB, and metalloproteinases, and to activate caspase 3. The former activities could contribute to its protective effect.

#### Butylated Hydroxytoluene

Butylated hydroxytoluene is a phenolic antioxidant and potent free radical scavenger that, like BHA, is a frequent food additive. It was moderately effective in the prevention of HC damage in the assay. It has not previously been studied as a HC protectant.

#### Dithiotreitol

Dithiotreitol is a thiol-containing, reducing agent that is a free radical scavenger and metal chelator. It was moderately protective in the assay. It has not previously been evaluated for its ability to protect HCs.

#### MC-186

MC-186 (MCI-186; edaravone) is a non-phenolic antioxidant. It is a potent free-radical and protein carbonyl scavenger and inhibitor of lipid peroxidation that is used in clinical trials for the treatment of chronic obstructive pulmonary disorder. It was moderately protective against high-dose gentamicin damage to HCs. Several previous studies have shown MC-186 to be protective against various forms of HC damage (e.g., Maetani et al., 2003; Horiike et al., 2004; Takemoto et al., 2004; Masuda et al., 2006).

### Procysteine

Procysteine (L-2-Oxothiazolidine-4-carboxylic acid, OTC) is a glutathione precursor that promotes rapid restoration of intracellular glutathione levels following depletion by ROS. Procysteine has been shown to reduce threshold shifts and HC loss following noise exposure in guinea pigs (Yamasoba et al., 1998), and to protect HCs in zebrafish from ototoxins (Ton and Parng, 2005).

#### Trolox

Trolox is a water-soluble, short chain vitamin E analog. It is a potent free radical scavenger. It was modestly protective in the assay. It has previously been shown to slightly inhibit HC damage due to gentamicin *in vitro* (Garetz et al., 1994b) as well as cisplatin- (Teranishi and Nakashima, 2003) and noise-induced (Yamashita et al., 2005) cochlear damage *in vivo*.

#### Thiourea

Thiourea is a thiol-containing reducing agent and free radical scavenger. It was modestly protective in the assay. Thiourea is toxic when administered at high systemic dosages. It preferentially inhibits the peroxidase in the thyroid gland and thus inhibits thyroxine production. The reduced synthesis of thyroid hormone causes an increased pituitary secretion of thyreotropic hormone and so hyperplasia of the thyroid which, on continuous stimulation in animals, can lead to tumor formation and, in man, to various thyroid-treated illnesses (Peters et al., 1949). However, intracochlear thiourea has been found to protect against cisplatin-induced HC loss *in vivo* (Ekborn et al., 2003).

#### Thymoquinone

Thymoquinone is a quinone antioxidant. It was minimally effective in our assay, showing protection only after 24 h of gentamicin treatment, and only at the lowest dosage. It is therefore possible that this represents a false positive. However, thymoqunone has previously been shown to protect HCs against damage due to gentamicin (Sagit et al., 2014), cisplatin (Sagit et al., 2013), and noise (Aksoy et al., 2015) *in vivo*.

It should be noted that some of the antioxidants that failed to exhibit protection have been shown in previous studies to protect HCs and hearing. Consequently, their failure to provide protection in our assay was unanticipated and surprising. Nonetheless, there are many potential explanations for these differences. They may be related to the relatively high concentration of gentamicin employed in our assay (200 μM), which presumably provided a very strong oxidative stress response. Differences could also be related to the *in vitro* nature of our assay, specific culture conditions, redox compound effective concentrations, species differences between mice and other animal models or humans, or to the use of neonatal as opposed to adult HCs.

### Antioxidants as HC Protectants

The range of protective and toxic responses observed in the assay illustrates the complexity of antioxidative compounds and their underlying mechanisms. Antioxidant effectiveness depends on several important intrinsic factors: permeability, activation energy, rate constants, molecular stability, oxidation–reduction potential, and solubility (Nawar, 1996). These functional factors are in turn closely related to the antioxidant molecular structure. Those antioxidants capable of interrupting the free radical chain reaction are usually the most effective (Brewer, 2011). They are characterized by aromatic or phenolic rings and act by donating a hydrogen atom to free radicals formed during oxidation. In the process, they transition into a radical form themselves; however, these radical intermediates are stable due to resonance delocalization of the extra electron within the aromatic ring and subsequent formation of stable quinones (Nawar, 1996). The most effective phenolic antioxidants have low oxidation-reduction potentials and OH groups in the *ortho*-position on the B phenolic ring. Phenolic antioxidants were well represented in our protective antioxidants. However, our two most effective antioxidants were quinone antioxidants. While they both possess an aromatic ring, the rings themselves are not characterized by OH groups, which lie elsewhere on the molecule.

As noted above, antioxidants also vary considerably in their mechanisms. These include scavenging the species that initiate peroxidation, quenching singlet oxygen, metal chelating, interrupting free radical chain reactions, and reducing oxygen concentrations (Badarinath et al., 2010). Antioxidants are not all equally powerful in reacting according to these varied mechanisms. For example, phenolic acids effectively trap free radicals but are not efficient metal chelators, while flavonoids can both scavenge free radicals and chelate metals efficiently (Brewer, 2011). Antioxidants can also differ in their ability to access the intracellular environment, and exhibit different half-lives. Finally, as noted above for some of the protective compounds, antioxidants can also have effects on cells that are not related to their antioxidant properties. This complexity makes the choice of antioxidants to employ difficult. The value of a broad screen, as employed here, is that no *a priori* knowledge of the properties of an antioxidant are required, and unexpected results may be obtained.

## Author Contributions

VN, AK, and AR designed, performed the experiments and wrote the manuscript. VN, KP, and RJ performed the studies.

## Conflict of Interest Statement

AR is a co-founder of, shareholder in and consultant to Otonomy, Inc. which develops slow-release therapeutics for the treatment of ear disease. This relationship has been approved by the Committee on Conflict of Interest at UCSD. The company played no role in this research. The other authors declare that the research was conducted in the absence of any commercial or financial relationships that could be construed as a potential conflict of interest.

## Abbreviations

BHA: butylated hydroxyanisole
BHT: butylated hydroxytoluene
DTT: dithiotreitol
GFP: green florescent protein
HCs: hair cells
NAC: *N*-acetyl cysteine
NFκB: nuclear factor kappa B
oC: organ of Corti
OH: hydroxyl
ROS: reactive oxygen species
