# Supplementary material for: An Antioxidant Screen Identifies Candidates for Protection of Cochlear Hair Cells from Gentamicin Toxicity
Source: Front Cell Neurosci. 2017 Aug 18;11:242. doi: 10.3389/fncel.2017.00242 (PMC5563352; doi:10.3389/fncel.2017.00242)
Supplement: Supplementary file 1 [file Table_1.PDF]

Supplementary Table 1. The inhibitors present in the Redox Library.

No.	Plate Location	Compound	Activity
1	1-A1	Promethazine·HCl	Secondary anti-oxidant
2	1-A2	Cumene hydroperoxide	Aryl hydroperoxide
3	1-A3	β-Lapachone	Undergoes futile redox cycles
4	1-A4	Resveratrol	Stilbene phenolic antioxidant
5	1-A5	Hydroquinone	Phenolic antioxidant
6	1-A6	TEMPOL	SOD mimetic
7	1-A7	Ferulic acid ethylester	Phenolic antioxidant
8	1-A8	D-α-Tocopherylquinone	Oxidized vitamin E
9	1-A9	Seratrodast	Quinone antioxidant
10	1-A10	Idebenone	Quinone antioxidant
11	1-A11	tert-Butylhydroquinone	Phenolic antioxidant
12	1-A12	HBED·HCl·H_2O	Metal chelator
13	1-B1	Ambroxol	Nonphenolic antioxidant
14	1-B2	L-Ergothioneine	Endogenous antioxidant
15	1-B3	Hinokitiol	Metal chelator
16	1-B4	Epigallocatechin gallate	Polyphenol
17	1-B5	Procysteine	Glutathione precursor
18	1-B6	Trolox	Short-chain vitamin E analog
19	1-B7	MCI-186	Nonphenolic antioxidant
20	1-B8	U83836E·2HCl	Antioxidant Lazaroid
21	1-B9	U74389G maleate	Antioxidant Lazaroid
22	1-B10	GERI-BP002A	Phenolic antioxidant
23	1-B11	Apigenin	Flavone antioxidant
24	1-B12	Terbinafine·HCl	Free radical quencher
25	1-C1	Rosmarinic acid	Phenolic antioxidant
26	1-C2	Piceatannol	Stilbene phenolic antioxidant
27	1-C3	AA-861	Quinone antioxidant
28	1-C4	CDC	Phenolic antioxidant
29	1-C5	Ebselen	Glutathione peroxidase mimetic
30	1-C6	Genistein	Isoflavone antioxidant
31	1-C7	Curcumin	Phenolic antioxidant
32	1-C8	Phenidone	Nonphenolic antioxidant
33	1-C9	Gossypol	Phenolic antioxidant
34	1-C10	Gentisic acid	Phenolic antioxidant
35	1-C11	Caffeic acid	Phenolic antioxidant
36	1-C12	Baicalein	Flavone antioxidant
37	1-D1	Esculetin	Coumarin antioxidant
38	1-D2	N-Propyl gallate	Phenolic antioxidant
39	1-D3	ETYA	Acetylenic antioxidant
40	1-D4	CAPE	Phenolic antioxidant
41	1-D5	NDGA	Phenolic antioxidant
42	1-D6	Capsaicin	Phenolic antioxidant
43	1-D7	BHT	Phenolic antioxidant
44	1-D8	BHA	Phenolic antioxidant
45	1-D9	Bakuchiol	Phenolic antioxidant
46	1-D10	DL-α-Lipoic acid	Sulfur-containing antioxidant
47	1-D11	Eugenol	Phenolic antioxidant
48	1-D12	Melatonin	Nonphenolic antioxidant
49	1-E1	N-Acetyl-Cysteine	Thiol-containing reducing agent
50	1-E2	D-γ-Tocopherol	Phenolic antioxidant
51	1-E3	Tocopherol succinate	Phenolic antioxidant
52	1-E4	Ascorbic acid	Ascorbate-type antioxidant
53	1-E5	Ascorbyl palmitate	Lipohilic ascorbate
54	1-E6	n-Octyl caffeate	Phenolic antioxidant
55	1-E7	Paeonol	Phenolic antioxidant
56	1-E8	Protocatechuic acid	Phenolic antioxidant
57	1-E9	Glutathione	Thiol-containing reducing agent
58	1-E10	Carvedilol	Nonphenolic antioxidant
59	1-E11	Diludin	Dihydropyridine-type antioxidant
60	1-E12	Carnosic acid	Phenolic antioxidant
61	1-F1	Tanshinone IIA	1,2-Quinone antioxidant
62	1-F2	Probucol	Phenolic antioxidant
63	1-F3	EPA	Polyunsaturated radical scavenger
64	1-F4	DCHA	Polyunsaturated radical scavenger
65	1-F5	bis-demethoxycurcumin	Phenolic antioxidant
66	1-F6	Ibuproxam	Metal chelator
67	1-F7	Ciclopirox ethanolamine	Hydroxyl radical scavenger
68	1-F8	Thymoquinone	Quinone antioxidant
69	1-F9	Thiourea	Thiol-containing reducing agent
70	1-F10	DTT	Thiol-containing reducing agent
71	1-F11	N-Ethylmaleimide	Thiol trap
72	1-F12	Buthionine sulfoximine	Glutathione biosynthesis inhibitor
73	1-G1	Anethole trithione	Sulfur-containing antioxidant
74	1-G2	TEMPO	Nitroxyl radical
75	1-G3	D609	Antioxidant
76	1-G4	Captopril	Thiol-containing reducing agent
77	1-G5	Disulfiram	Sulfur-containing antioxidant
78	1-G6	1,2-Dithiole-3-thione	Sulfur-containing antioxidant
79	1-G7	Selenomethionine	Selenium-containing antioxidant
80	1-G8	Tetramethylpyrazine	Nonphenolic antioxidant
81	1-G9	Ethoxyquin	Nonphenolic antioxidant
82	1-G10	Canthaxanthin	Polyunsaturated radical scavenger
83	1-G11	β-carotene	Polyunsaturated radical scavenger
84	1-G12	Retinyl palmitate	Polyunsaturated radical scavenger
